# Prognostic significance of p53 expression in patients with esophageal cancer: a meta-analysis

**DOI:** 10.1186/s12885-016-2427-6

**Published:** 2016-07-01

**Authors:** Lianghai Wang, Xiaodan Yu, Jing Li, Zhiyu Zhang, Jun Hou, Feng Li

**Affiliations:** Department of Pathology and Key Laboratories for Xinjiang Endemic and Ethnic Diseases, Shihezi University School of Medicine, Shihezi, Xinjiang China; Department of Immunology, Shihezi University School of Medicine, Shihezi, Xinjiang China; Department of Pathology, Beijing Chaoyang Hospital, Capital Medical University, Beijing, China

**Keywords:** p53, Esophageal cancer, Prognosis, Meta-analysis

## Abstract

**Background:**

The prognostic value of p53 protein expression in esophageal cancer has been evaluated, but the results remain inconclusive and no consensus has yet been achieved. This meta-analysis was conducted to quantitatively assess the prognostic significance of p53 expression in esophageal cancer.

**Methods:**

Publications that assessed the clinical or prognostic significance of p53 expression in esophageal cancer and were published before July 1, 2015 were identified by searching the PubMed and EMBASE databases. A meta-analysis was performed to clarify the association between p53 expression and the clinical outcomes.

**Results:**

A total of 36 publications met the criteria and included 4577 cases. Analysis of these data showed that p53 expression in esophageal cancer was significantly associated with poorer 5-year survival (RR = 1.30, 95 % CI: 1.11–1.51, *P* = 0.0008). Subgroup analyses according to histological type, continent of the patients, and cut-off value revealed the similar results. The results also indicated that p53 expression was highly associated with advanced TNM stages (I/II vs. III/IV, OR = 0.74, 95 % CI: 0.55–0.99, *P* = 0.04), lymph node metastasis (OR = 0.77, 95 % CI: 0.66–0.90, *P* = 0.001), and distant metastasis (OR = 0.46, 95 % CI: 0.26–0.80, *P* = 0.006). However, p53 expression in the included studies was not significantly associated with tumor size (≤ 5 cm vs. > 5 cm, OR = 1.13, 95 % CI: 0.92–1.40, *P* = 0.24), tumor location (upper + middle vs. lower, OR = 0.91, 95 % CI: 0.70–1.17, *P* = 0.45), grade of differentiation (well + moderate vs. poor, OR = 1.10, 95 % CI: 0.90–1.34, *P* = 0.35), and the depth of invasion (T1/T2 vs. T3/T4, OR = 0.86, 95 % CI: 0.71–1.03, *P* = 0.09).

**Conclusions:**

This meta-analysis showed that p53 expression may be a useful biomarker for predicting poorer prognosis in patients with esophageal cancer.

## Background

Esophageal cancer (EC), a highly aggressive and lethal malignancy, causes 400 200 deaths worldwide and is the sixth leading cause of cancer death in 2012 [1]. This malignancy includes two major histological types: esophageal squamous cell carcinoma (ESCC) and esophageal adenocarcinoma (EAC). Although the relevant diagnosis and treatment methods have dramatically improved in recent years, atypical early symptoms, middle-to-late stage diagnosis, low treatment remission rates, and high local recurrence rates continue to contribute to the poor prognosis of patients with EC [2]. The increasing incidence and poor prognosis of EC represent a major global public health problem [3]. Despite advancements in diagnostic and treatment methods in recent years, the prognosis of patients with EC remains not ideal. Only a small group of patients (15–30 %) survive five years after surgery [4, 5]. Therefore, the detailed molecular mechanisms involved in EC progression must be understood and prognostic factors should be identified to enable the precise prediction of survival and selection of better treatment and preventive measures for patients with EC.

A few biomarkers, including p53, vascular endothelial growth factor (VEGF) [6], and CXC chemokine receptor type 4 (CXCR4) [7], have recently emerged as prognostic or predictive factors in EC. *p53*, a tumor-suppressor gene, is located on the short arm of chromosome 17 and displays the highest correlation with human types of cancer uncovered thus far. This gene encodes the p53 protein, which acts as a transcription factor that plays a key role in cell cycle regulation, DNA synthesis inhibition, damaged DNA repair, and apoptosis [8, 9]. Under normal conditions, p53 levels are low; in some cases, they may even be undetectable [10]. However, the expression rate of p53 detected by immunohistochemistry (IHC) has been reported to range from 33 to 70 % in EC [11, 12]. p53 shows nuclear staining because of accumulation of mutant p53, which often has an increased stability and is resistant to degradation, making it detectable by IHC [13]. A cell without mutation is negative for IHC staining of p53 because no dye accumulation occurs in the cell [14]. Although accumulation of p53 detected by IHC does not necessarily imply gene mutation, p53 over-expression in most of cases (85 %) implies an underlying mutation [15]. Therefore, p53 expression may be regarded as an indicator of *p53* gene mutation.

Over the past decade, numerous studies have evaluated the prognostic value of p53 protein expression in EC. However, the results of these reports remain inconclusive and no consensus has yet been achieved. Therefore, we conducted a systematic review and meta-analysis to address the association between p53 expression and the common clinical and pathological features of EC.

## Methods

### Search strategy

We considered all studies on the association between p53 and EC in this research. A systematic search was performed with the following keywords or their combinations: “p53” or “TP53” and “esophageal cancer” or “esophageal carcinoma.” The search was performed in the PubMed and EMBASE databases. The last search in this study was updated in July 2015.

### Inclusion criteria

All of the original studies must meet the following criteria to be included in this meta-analysis: (1) Patients were confirmed as EC by pathological examination. (2) The expression of p53 in primary tumor tissues was detected by IHC. (3) None of patients had received radiation therapy or chemotherapy before surgery. (4) The sample size was greater than 20. (5) The association between p53 expression and overall survival (OS) of the patients with EC was evaluated. (6) Sufficient data were provided to allow the estimation of risk ratios (RRs) or odds ratios (ORs) and their corresponding 95 % confidence intervals (CI). (7) Only studies written in English and Chinese were included in this study.

### Exclusion criteria

The search was broadened by browsing the related summary, methods, and references of retrieved articles. The title and abstract of each study identified in the search were scanned to exclude clearly irrelevant publications. The remaining articles were browsed to determine whether they contained information on the topic of interest. We excluded studies from this meta-analysis if they were: (1) review articles, case reports, familiar studies, duplicated publications, conference abstracts, and letters; (2) studies where p53 expression was evaluated by a method other than IHC; (3) studies with sample sizes less than 20; (4) studies without clinical data and the relationship between p53 expression and disease prognosis; (5) duplicate articles. For duplicate studies based on identical or overlapping patient populations, only the most recent and/or complete study was included in this meta-analysis.

### Data extraction

Information was carefully and independently extracted from all eligible publications by two of the authors according to the inclusion criteria listed above. Disagreement was resolved by discussion between the two authors until a consensus was reached. Data tables were constructed to extract all relevant data from the text, tables, and figures of each included study, including the author, publication year, country of patient’s origin, tumor stage, number of patients, research technique, and cut-off value of p53 expression. When the prognosis was only plotted as a Kaplan–Meier curve in some articles, Engauge Digitizer 4.1 software (from https://sourceforge.net/projects/digitizer/) was applied to digitize and extract the data.

### Statistical analysis

ORs with 95 % CI were used to evaluate the association between p53 expression and clinicopathological factors, including the tumor TNM stage, tumor size, tumor location, grade of differentiation, depth of invasion, lymph node involvement, and distant metastasis. To stratify data for analysis, the p53 expression and clinicopathological factors were combined into single categories with comparable clinicopathological relevance: tumor TNM staging (I/II vs. III/IV), lymph node (negative or positive), distant metastasis (negative or positive), tumor size (≤ 5 cm vs. > 5 cm), tumor location (upper + middle vs. lower), grade of differentiation (well + moderate vs. poor), and depth of invasion (T1/T2 vs. T3/T4). RRs with 95 % CI were used to assess the association between p53 expression and the combined survival outcome over several studies. The presence of heterogeneity among studies was evaluated by the Dersimonian and Laird’s Q test. I^2^ was used to quantify heterogeneity, and an I^2^ value > 50 % was considered to represent substantial heterogeneity between studies [16]. Compared with fixed-effects models, random-effects models were found to be more appropriate for the current study because of the heterogeneity revealed by the forest plots. Heterogeneity often cannot be revealed by the Q test because of its low power. The influence of individual studies on the estimated summary effect was displayed in the sensitivity analysis. In addition, funnel plots were used to estimate the possible publication bias. Cochrane Review Manager version 5.2 (Cochrane Library) was used to calculate the ORs and RRs, as well as their variations, from each investigation.

## Results

### Description of studies

**Fig. 1:**
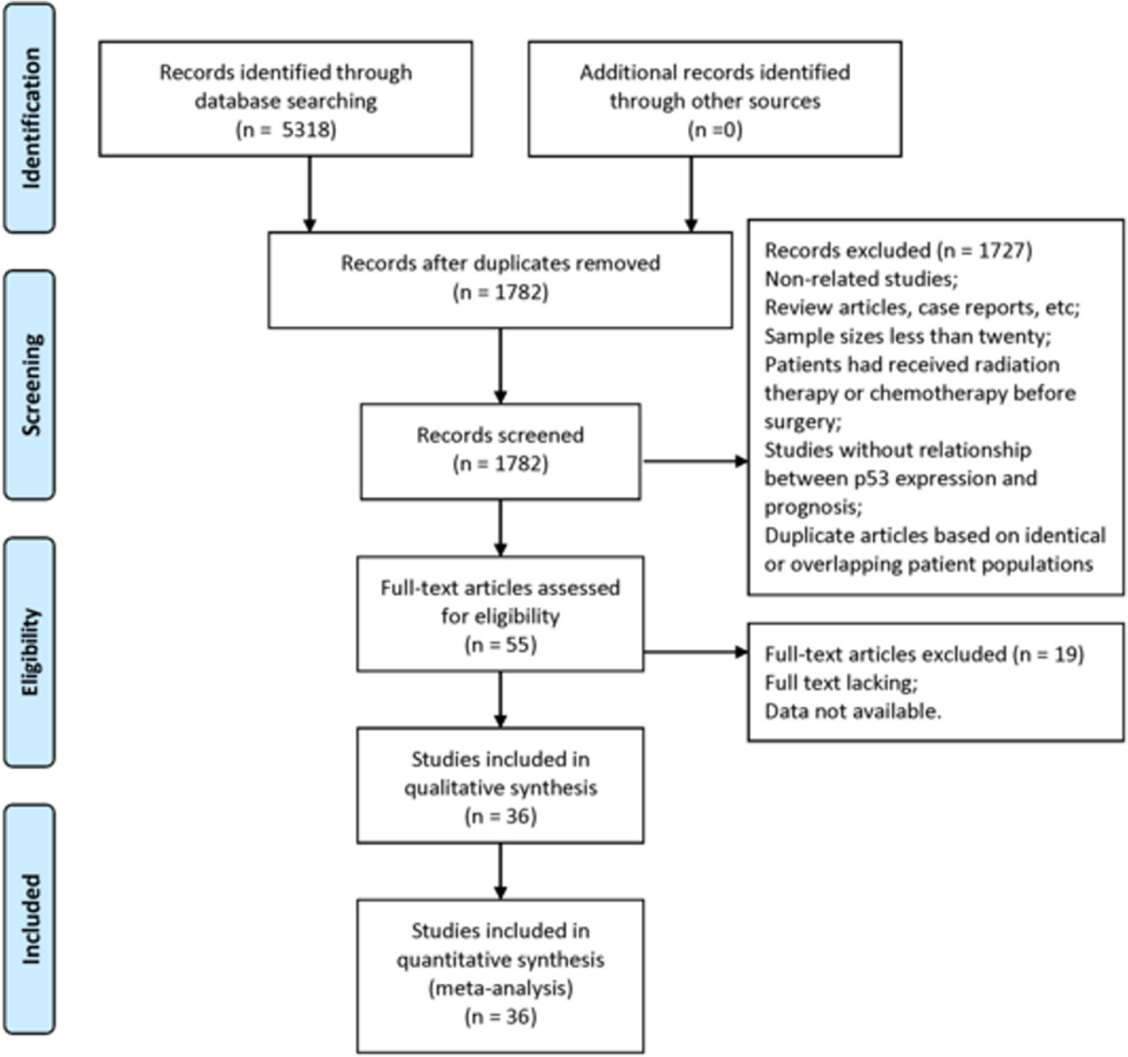
Literature search strategy and selection of articles

A total of 36 publications met the criteria for the analysis (Fig. 1). The total number of patients was 4577, with 33–775 patients per study. The main characteristics of the eligible studies, including the cut-off definition for p53-positive results, are summarized in Table 1. All of the studies determined the OS, and some reports included clinicopathological factors. IHC was the only method used to evaluate p53 expression in EC specimens.

**Table 1:** Characteristics of studies included in this meta-analysis

Study	Country	Type	Source	Cases	p53 positive rate (%)	IHC Cut off (nuclear positivity)
Madani K, 2010 [12]	Canada	EAC	esophagectomy	142	33.8	>10 %
Casson AG, 1998 [28]	Canada	ESCC/EAC	esophagectomy	61	39	>10 %
Rosa AR, 2003 [29]	Brazil	ESCC	esophagectomy	47	53.2	>10 %
Bahnassy AA, 2005 [30]	Egypt	ESCC/UC	esophagectomy	50	68	>10 %
Egashira A, 2011 [31]	Japan	ESCC	esophagectomy	94	56.4	>10 %
Chanvitan A, 1995 [32]	Canada	ESCC	esophagectomy	80	50	>10 %
Murata, A, 2013 [33]	Japan	ESCC	esophagectomy	266	52	weak-to-strong
Wang DY, 1994 [34]	China	ESCC	esophagectomy	100	65	>30 %
Kato H, 2001 [35]	Japan	ESCC	esophagectomy	89	55.1	>10 %
Flejou JF, 1994 [36]	France	EAC	esophagectomy	62	66	ND
Shimaya K, 1993 [37]	Japan	ESCC	esophagectomy	105	53	any nuclear positivity
Huang K, 2014 [38]	China	ESCC	esophagectomy	118	49.2	>10 %
Lam KY, 1999 [39]	China	ESCC	esophagectomy	153	64.1	>25 %
Chyczewski L, 1999 [40]	Poland	ESCC	esophagectomy	33	45	>10 %
Cavazzola LT, 2009 [41]	Brazil	EAC	esophagectomy	38	52.2	>10 %
Shang L, 2014 [42]	China	ESCC	esophagectomy	590	43	>10 %
Yasuda M, 2000 [27]	Japan	EC	esophagectomy	35	48.5	dark brown
Kuwahara M, 1999 [43]	Japan	EC	esophagectomy	64	48.4	>10 %
Nita ME, 1999 [44]	Brazil	ESCC	esophagectomy	62	50	>10 %
Ikeguchi M, 2000 [45]	Japan	ESCC	esophagectomy	191	44.5	>50 %
Furihata M, 1993 [46]	Japan	ESCC	esophagectomy	71	33.8	ND
Ahn MJ, 2002 [47]	Korea	ESCC/BSCC	esophagectomy	81	51.9	>10 %
Hashimoto N, 1999 [48]	Japan	ESCC	esophagectomy	73	64	>5 %
Makoto O, 2002 [49]	Japan	ESCC	esophagectomy	96	46	>10 %
Hsu PK, 2008 [50]	China	ESCC	esophagectomy	68	63.2	>25 %
Kanamoto A,1999 [51]	Japan	ESCC	esophagectomy	239	48.1	>10 %
Hardwick RH, 1997 [52]	UK	ESCC/EAC	esophagectomy	78	66.7	>10 %
Vijeyasingam R, 1994 [53]	England	ESCC/EAC	esophagectomy	60	68.3	>5 %
Inada S, 1999 [54]	Japan	ESCC	esophagectomy	40	52.5	>10 %
Nakamura T, 1995 [55]	Japan	ESCC	esophagectomy	61	52	ND
Cheng TH, 2009 [56]	China	ESCC	esophagectomy	119	51.3	>10 %
Yao W, 2014 [57]	China	ESCC	esophagectomy or endoscopy	136	41.9	weak-to-strong
Takeno S, 2002 [58]	Germany	ESCC	esophagectomy	71	36.6	>10 %
Xu XL, 2014 [59]	China	ESCC	esophagectomy	775	35.9	>10 %
Takahashi Y, 2006 [60]	Japan	ESCC	esophagectomy	180	61.7	>10 %
Goukon Y, 1994 [61]	Japan	ESCC	esophagectomy	49	59	any nuclear positivity

*UC* undifferentiated carcinoma, *BSCC* basaloid squamous cell carcinoma, *ND* not documented

### Correlation of p53 expression with clinicopathological parameters

**Fig. 2:**
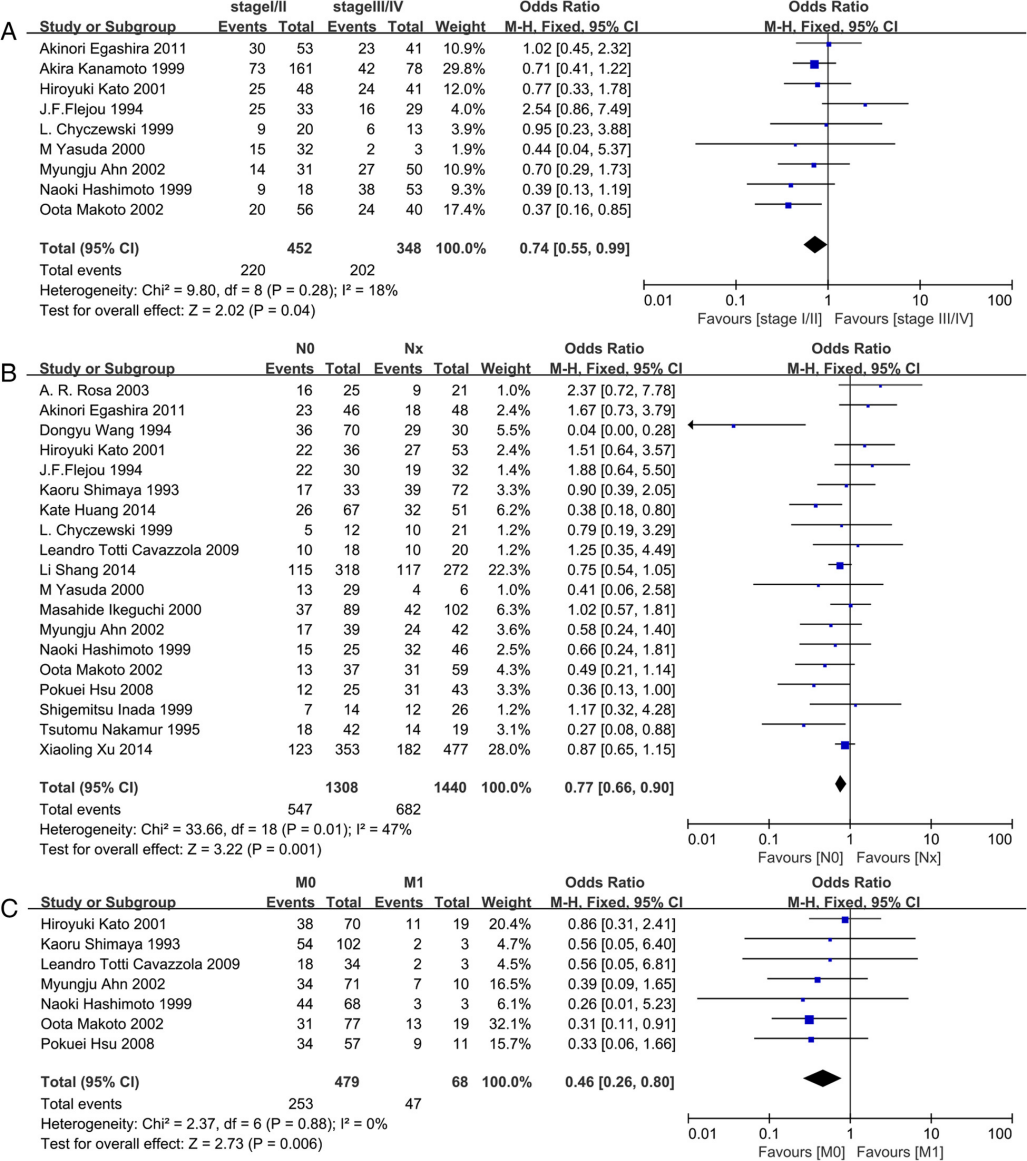
Forest plot of p53 expression and OR for clinicopathological features. The investigated clinicopathological parameters are TNM stage (**a**), lymph node metastasis (**b**), and distant metastasis (**c**). ORs with the corresponding confidence intervals are shown

The association between p53 and several clinicopathological parameters are illustrated in Fig. 2 and Table 2. The p53 expression was highly correlated with more advanced TNM stages (I/II vs. III/IV, OR = 0.74, 95 % CI: 0.55–0.99, *P* = 0.04, Fig. 2a), lymph node metastasis (OR = 0.77, 95 % CI: 0.66–0.90, *P* = 0.001, Fig. 2b), and distant metastasis (OR = 0.46, 95 % CI: 0.26–0.80, *P* = 0.006, Fig. 2c). However, p53 expression was not significantly associated with tumor size (≤ 5 cm vs. > 5 cm, OR = 1.13, 95 % CI: 0.92–1.40, *P* = 0.24), tumor location (upper + middle vs. lower, OR = 0.91, 95 % CI: 0.70–1.17, *P* = 0.45), grade of differentiation (well + moderate vs. poor, OR = 1.10, 95 % CI: 0.90–1.34, *P* = 0.35), and depth of invasion (T1/T2 vs. T3/T4, OR = 0.86, 95 % CI: 0.71–1.03, *P* = 0.09; Table 2).

**Table 2:** Meta-analysis of p53 expression and clinicopathological features of EC

Clinicopathological features	*N*	Cases	Analytical model	Pooled OR (95 % CI)	*P* value	Heterogeneity	
						I^2^ (%)	*P* value
Tumor size (≤ 5 cm vs. > 5 cm)	4	1515	FEM	1.13 (0.92–1.40)	0.24	0	0.96
Tumor location (upper + middle vs. lower)	8	1205	FEM	0.91 (0.70–1.17)	0.45	0	0.80
Grade of differentiation (well + moderate vs. poor)	16	2328	FEM	1.10 (0.90–1.34)	0.35	17	0.26
Depth of invasion (T1/T2 vs. T3/T4)	13	2262	FEM	0.86 (0.71–10.3)	0.09	0	0.67

*N* number of studies, *FEM* fixed-effect model

### p53 expression and five-year survival outcome

**Fig. 3:**
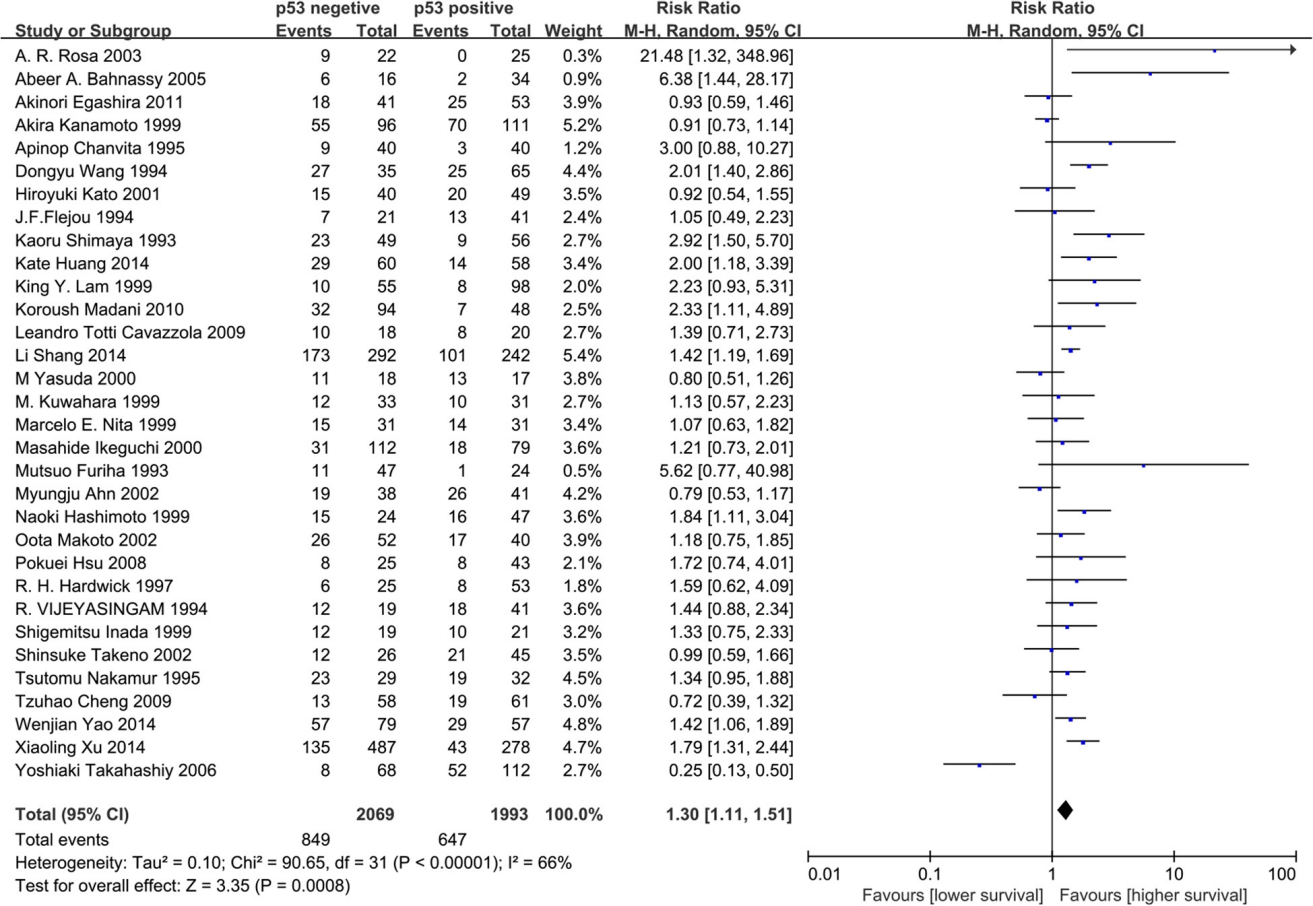
Analysis of p53 expression and survival of EC patients. Forest plot of RR for the OS included studies. Combined RR was calculated by a random model

Based on the methods described above, the OS of 4577 patients in 36 studies were analyzed. The 5-year OS rate was extracted from 32 studies. Meta-analysis of the 32 studies for the prognostic value of p53 expression showed that increased expression was associated with poorer OS. This trend was obtained from the M–H random-effects model with a value of 1.30 (95 % CI: 1.11–1.51, *P* = 0.0008; Fig. 3), although heterogeneity between studies was noted (I^2^ = 66 %, P_h_ < 0.00001).

Subgroup analyses were conducted to address the heterogeneity observed in the correlation between p53 overexpression and decreased OS in EC patients, according to histological type of EC, continent of the patients, and cut-off value of IHC (Table 3). Results showed the similar clinical significance of p53 expression in each of the two major histological types (pure ESCC cohorts: RR = 1.32, 95 % CI: 1.10–1.57, *P* = 0.002; pure EAC cohorts: RR = 1.61, 95 % CI: 1.05–2.47, *P* = 0.03). The association between p53 overexpression and poorer OS in EC patients appeared to be greater among studies involving patients from Europe and America (RR = 1.54, 95 % CI: 1.22–1.94, *P* = 0.0003) compared with studies involving patients from Asia (RR = 1.24, 95 % CI: 1.04–1.48, *P* = 0.02), and studies setting a none-10 % cut-off value (RR = 1.56, 95 % CI: 1.35–1.81, *P* <0.00001) compared with studies with a cut-off value of 10 % (RR = 1.18, 95 % CI: 0.96–1.45, *P* = 0.12).

**Table 3:** Subgroup meta-analyses of p53 expression and survival according to histological type, continent and cut-off value

Subgroup	*N*	Cases	Pooled RR (95 % CI)	*P* value	Analytical model	Heterogeneity	
						I^2^ (%)	*P* value
Histological type							
ESCC only	23	3454	1.32 (1.10–1.57)	0.002	REM	70	< 0.00001
EAC only	3	242	1.61 (1.05–2.47)	0.03	FEM	16	0.3
Continent							
Asia	22	3372	1.24 (1.04–1.48)	0.02	REM	72	< 0.00001
Europe and America	9	640	1.54 (1.22–1.94)	0.0003	FEM	30	0.18
Cut-off value							
10 %	20	2949	1.18 (0.96–1.45)	0.12	REM	71	< 0.00001
None-10 %	12	1113	1.56 (1.35–1.81)	< 0.00001	FEM	44	0.05

*N* number of studies, *FEM* fixed-effect model, *REM* random-effect model

### Sensitivity analysis

To test for bias introduced by the low number of available eligible publications, we performed a sensitivity analysis. A single study in the meta-analysis was omitted from each round of analysis to investigate the influence of the individual data set of a particular study on the pooled ORs. We found that the corresponding pooled ORs were not essentially altered by the subtraction of any study (data not shown), thereby indicating that our results were statistically robust.

### Publication bias

**Fig. 4:**
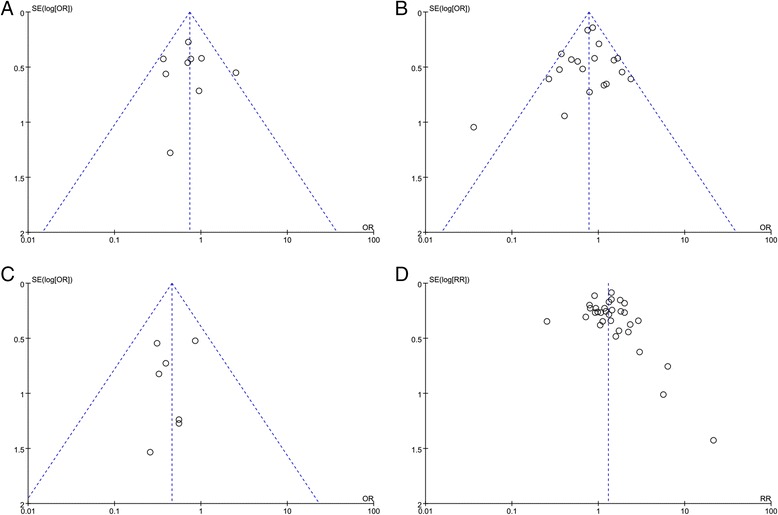
Publication bias determination using funnel plot. Funnel plots of TNM stage (**a**), lymph node metastasis (**b**), distant metastasis (**c**), and 5-year survival (**d**)

Funnel plots were performed to assess the publication bias in this meta-analysis. The shape of the funnel plots did not reveal obvious evidence of asymmetry (Fig. 4).

## Discussion

The clinical significance and prognostic value of p53 expression in EC has recently been reported by several investigators. In the present meta-analysis, we assess the association between p53 expression and survival, as well as the clinicopathological features in EC. A total of 36 relevant studies comprised of 4577 cases were subjected to the final analysis.

The tumor suppressor gene *p53* and its wild-type protein play multiple functions in regulating cell cycle progression, apoptosis, autophagy, differentiation, senescence, and DNA repair functions, as well as influences cell metabolic pathways and cytokines [17]. However, if *p53* is mutated, the mutant p53 protein can accumulate in the cell nucleus [18], although in some cases, nonsense mutations or a quickly degraded mutant protein can cause lack of expression [13]. Therefore, p53 over-expression is generally associated with the inactivation of p53 [19]. Based on its functions, positive p53 expression in cancer cells may promote cell migration, invasion, and metastasis, finally leading to poor prognosis [20]. In human cancers, the *p53* gene is the most commonly mutated gene; positive expression of p53 has been correlated with the clinicopathological features and prognosis of breast cancer [21], bladder cancer [22], and other types of cancer.

The results of the overall pooled analysis in the present study on the association of p53 expression with survival in EC patients suggested that positive p53 expression was significantly related to poorer OS (RR = 1.30, 95 % CI: 1.11–1.51). These findings demonstrated the significance of p53 expression in the prognosis of patients with EC and agreed with the theoretical inference that patients with positive p53 expression, which is often cause by mutation, could have poorer clinical prognosis than those with negative p53 expression. The same results have been reported in the meta-analyses of gastric cancer [23], osteosarcoma [24], hepatocellular carcinoma [25], and other tumors.

We also analyzed the relationship between p53 and clinicopathological parameters; the results showed that p53 expression was significantly associated with more advanced TNM stages (I/II vs. III/IV, OR = 0.74, 95 % CI: 0.55–0.99), lymph node metastasis (OR = 0.77, 95 % CI: 0.66–0.90), and distant metastasis (OR = 0.46, 95 % CI: 0.26–0.80). Given that a more advanced TNM stage, positive lymph node metastasis, and distant metastasis are adverse prognostic features, the present results may explain why positive p53 expression is associated with poor 5-year survival in patients with EC. However, no significant associations were observed between p53 expression and tumor size, tumor location, grade of differentiation, and depth of invasion in this study.

The current study presented several limitations that should be considered. First, the heterogeneity across studies was high for some parameters of this disease. Therefore, even if the random-effects models are used to take heterogeneity into account and several heterogeneity analyses were performed, some estimates should be interpreted with caution. The second limitation involves the lacking of a defined standardized protocol and evaluation system to measure p53 expression by IHC in various studies; several factors, such as differences in types of antibodies, concentrations, and cut-off values used may lead to potential bias. Nevertheless, the sensitivity of IHC to assess p53 mutations through protein accumulation is generally poor; some mutations, such as truncated mutant, can lead to complete loss of p53 staining and be missed by IHC [13, 26, 27]. Combining IHC and other widely applicable techniques, which could detect p53 gene aberrations, would potentially improve the accuracy of p53 as a clinical biomarker for predicting EC progression. Third, the full text of studies in this meta-analysis were published only in English or Chinese. Non-significant or negative findings are usually not published and other potential eligible studies may have been excluded; these factors also contribute to bias. We included the data of 4577 patients in this meta-analysis to provide a foundation for a larger prospective study.

## Conclusions

In conclusion, our findings indicate that positive p53 expression is independently and significantly associated with poorer 5-year survival, more advanced TNM stages, lymph node metastasis, and distant metastasis in patients with EC. The expression of p53 may be a useful biomarker to predict a poorer prognosis for EC patients. However, to strengthen our findings, larger prospective studies with better standardized methods are needed to provide a comprehensive conclusion regarding the prognostic role of p53 expression in EC.

## Abbreviations

BSCC, basaloid squamous cell carcinoma; CI, confidence interval; EAC, esophageal adenocarcinoma; EC, esophageal cancer; ESCC, esophageal squamous cell carcinoma; FEM, fixed-effect model; IHC, immunohistochemistry; N, number of studies; ND, not documented; OR, odds ratio; OS, overall survival; RR, risk ratio; UC, undifferentiated carcinoma
